# Editorial: COVID-19 epidemiological situation as a psychosocial determinant of trauma and stress

**DOI:** 10.3389/fpsyg.2023.1352269

**Published:** 2024-01-10

**Authors:** Mateusz Grajek, Ewa Misterska, Jonathan Adedayo Odukoya, Mateusz Rozmiarek

**Affiliations:** ^1^Department of Public Health, Faculty of Public Health in Bytom, Silesian Medical University in Katowice, Katowice, Poland; ^2^Department of Pedagogy and Psychology, Faculty of Social Studies in Poznan, Poznan School of Security, Poznan, Poland; ^3^Department of Psychology, Covenant University, Ota, Nigeria; ^4^Department of Sports Tourism, Faculty of Physical Culture Sciences, Poznan University of Physical Education, Poznan, Poland

**Keywords:** COVID-19, SARS-CoV-2, stress, trauma, epidemiology, psychology, mental health

Infectious diseases such as COVID-19 (coronavirus disease), affecting the respiratory system, have been recognized as a global pandemic due to the rapid transmission of the SARS-CoV-2 virus. While the disease manifested mildly in the majority of individuals, some patients, particularly the elderly and/or those with underlying chronic comorbidities, experienced the development of severe bilateral pneumonia, acute respiratory distress syndrome, and consequently, multiorgan dysfunction, potentially leading to mortality (Yong, 2021; Merad et al., 2022). Consequently, the fear of infection, especially the severe course of the disease and death, undoubtedly became a cause of generalized anxiety and fear for many individuals (Gunnell et al., 2020). Frequently, the overall uncertainty stemming from the evolving societal situation became a predisposing factor for mood deterioration, worsened wellbeing, and diminished quality of life. The mentioned anxiety was driven not only by concerns for one's own health but also for the health and lives of close ones. The COVID-19 pandemic unquestionably had a profound impact on the mental health of society, leaving a lasting imprint. The fear of the unknown, specifically the announcement by the World Health Organization (WHO) on March 11, 2020, of a new coronavirus pandemic, contributed to the manifestation of anxiety symptoms in the population, thereby exacerbating mental health problems, even in initially healthy individuals (Botha et al., 2020; Li et al., 2020).

Global statistics related to COVID-19 can be deemed alarming, as since the beginning of the pandemic, ~680 million people worldwide have been infected with COVID-19, and nearly seven million have died. Due to the potential severity of the disease, the WHO has issued official information and recommendations to minimize the risk of infection from the onset of the first cases of COVID-19. The daily lives of many people have changed significantly, with numerous interpersonal relationships severed, leading to experiences of loneliness and social exclusion for many. The ongoing pandemic has posed a challenge not only to the healthcare sector but also to the entire global economy, as well as education, tourism, culture, and the broader field of public health in countries worldwide.

It has resulted in negative changes across various aspects of life, the consequences of which society continues to bear. The cumulative impact of these factors has a destructive influence on the mental health of society globally (Holmes et al., 2020).

All these aspects have contributed to the initiation of a Research Topic titled “*COVID-19 epidemiological situation as a psychosocial determinant of trauma and stress*,” comprising 11 original articles, one conceptual analysis, and one brief research report dedicated to this subject.

The article *Resilience mediates the effect of the COVID-19 pandemic on mental health in a sample of adults in Panama* by Oviedo et al. details the mediating role of resilience in the relationship between perceived COVID-19 impact and mental health symptoms. Individuals more personally affected by the pandemic were found to exhibit heightened depression, anxiety, and stress symptoms due to diminished resilience. Simultaneously, the article *Lived experience of Iranian pre-hospital medical staff during the COVID-19 pandemic: a descriptive phenomenological study* by Jafari-Oori et al. sheds light on the challenges faced by pre-hospital medical staff in Iran, stemming from inadequate preparedness and substantial adversity during the pandemic. Additionally, *The impacts of the COVID-19 pandemic on indirect costs of mental illness and behavioral disorders in Poland* by Sobczyk et al. marks the inception of a study investigating the economic burden of COVID-19 indirect costs in the country. Moreover, the article *Emotional control and factors differentiating it in the adult population of Poland during the COVID-19 pandemic* by Głogowska-Gruszka and Wypych-Ślusarska discloses that a higher level of knowledge about the pandemic and preventive measures correlates with increased emotional control, particularly in the anxiety subscale.

The longitudinal mixed-methods study titled *Older adults' coping strategies during the COVID-19 pandemic—a longitudinal mixed-methods study* by Kastner et al. provides valuable insights into the interplay of personal prerequisites, pandemic assessment, and coping strategies, utilizing an adapted Lazarus stress model. Simultaneously, the article *Mental health in Canadian children and adolescents during COVID-19 pandemic: the role of personality and, coping and stress responses* by Shokrkon and Nicoladis uncovers an association between personality traits and the mental health of Canadian youth amidst the pandemic. Furthermore, the qualitative study *Challenges to dialysis treatment during the COVID-19 pandemic: a qualitative study of patients' and experts' perspectives* by Oviedo Flores et al. highlights concerns among hemodialysis patients, including being “high-risk” and preferences for home dialysis over in-center dialysis, while healthcare professionals emphasize the impact of changes in clinical routine and the emergence of telehealth. Additionally, the article *Impact of COVID-19 on employment*: *sociodemographic, medical, psychiatric and neuropsychological correlates* by Thompson et al. demonstrates significant variations between TTO vs. NTO and PS vs. PDNS in medical, psychiatric, and neurocognitive domains, and subjective measures of symptoms, providing a comprehensive understanding of the multifaceted impact of the pandemic.

The article *How COVID-19 pandemic period influences on the selected mental health parameters of Polish respondents?* By Florek et al. delves into the positive correlations between anxiety and various forms of aggression within the study population, highlighting distinctions in these associations across genders, age groups, and educational backgrounds. Simultaneously, the article *Development and psychometric properties of health care workers' concerns in infectious outbreaks scale* by Yarahmadi et al. introduces the 36-item Health Care Workers' Concerns in Infectious Outbreaks Scale (HCWCIOS) with robust psychometric properties, making it suitable for evaluating healthcare workers' concerns during a pandemic. Furthermore, the article *Using knowledge of, attitude toward, and daily preventive practices for COVID-19 to predict the level of post-traumatic stress and vaccine acceptance among adults in Hong Kong* by Cao et al. posits that individuals exhibiting good preventive practices, limited knowledge, and negative attitudes toward COVID-19 are more prone to post-traumatic stress disorder. Conversely, a positive attitude, coupled with adherence to preventive practices, significantly predicts willingness to receive vaccination and engage in voluntary testing.

The conceptual analysis article titled *Helper Syndrome and Pathological Altruism in nurses – a study in times of the COVID-19 pandemic* by Maringgele et al. unveils groundbreaking findings by illustrating Schmidbauer's concept of Helper Syndrome for the first time. Notably, the data pointed toward the presence of a subgroup aligning with Schmidbauer's Helper Syndrome description, independent of their professional roles in helping or non-helping capacities. Crucially, individuals within this subgroup appeared to be at increased risk of psychiatric disorders. Additionally, the brief research report titled *Social value of pathology: adapting primary health care to reduce stress and social anxiety in college students exposed to social distancing* by Sava discloses that educational content, delivery methods, increased homework, and extended online engagement potentially contributed to heightened stress, depression, and social anxiety disorder levels in approximately one-third of students engaging in digital learning.

The results presented in the articles and the derived conclusions constitute a significant contribution to the identification and monitoring of psychosocial issues currently faced by society in the post-COVID-19 pandemic era. In accordance with the WHO definition, mental health determines an individual's capacity for continuous development and self-realization. As indicated by the findings of the conducted research, the period from the onset of the COVID-19 pandemic until the lifting of associated sanitary restrictions represents a highly traumatizing and developmentally inhibiting phase for both individual and societal growth. The effects of misinformation, mandates/prohibitions, isolation, experienced grief, and the resultant high levels of stress and negative emotions are evident, among other aspects, in outcomes related to worsened wellbeing, heightened anxiety and fear, and the occurrence of depressive symptoms. However, further research on this matter is still necessary to diagnose the effects of the COVID-19 pandemic on society in an even more precise manner (Jin et al., 2021; Meherali et al., 2021; Riedel et al., 2021; Weich, 2022).

The experience of the COVID-19 pandemic should lead to the introduction of measures to nullify and minimize the subsequent negative effects on the mental health of the population by organizing coordinated efforts at multiple legislative and executive levels that would lead to the identification of symptoms and the reduction of negative effects on the mental health of individuals (Figure 1).

**Figure 1:**
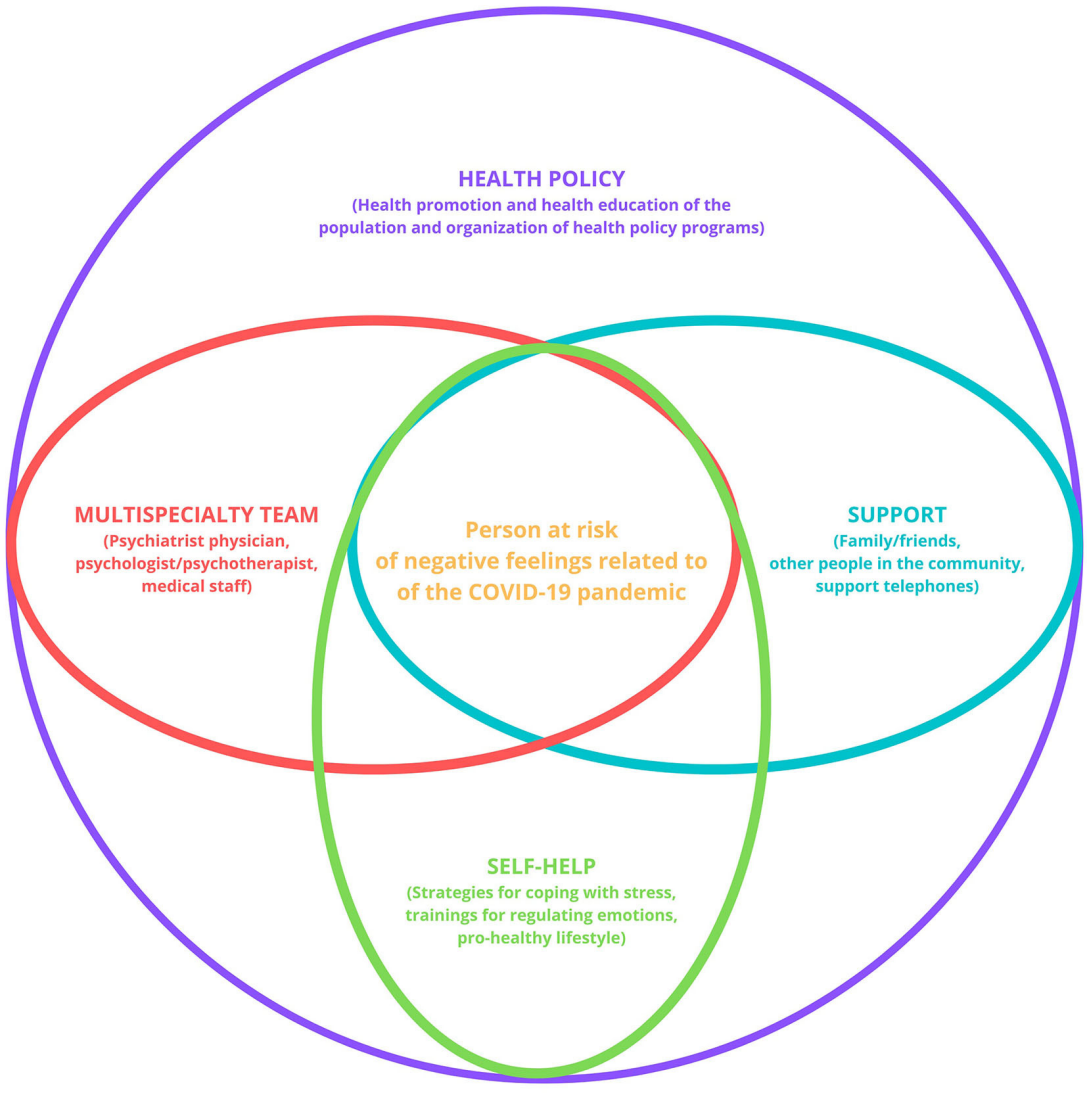
Unified system of complex support for people at risk of negative feelings related to the COVID-19 pandemic. Source: own work.

## Author contributions

MG: Conceptualization, Writing – original draft. EM: Resources, Writing – review & editing. JO: Resources, Writing – review & editing. MR: Conceptualization, Visualization, Writing – review & editing.
